# LMT^db^: A comprehensive transcriptome database for climate-resilient, nutritionally rich little millet (*Panicum sumatrense*)

**DOI:** 10.3389/fpls.2023.1106104

**Published:** 2023-03-09

**Authors:** Shweta Shekhar, Archana S. Prasad, Kalpana Banjare, Abhijeet Kaushik, Ajit K. Mannade, Mahima Dubey, Arun Patil, Vinay Premi, Ashish K. Vishwakarma, Abhinav Sao, Ravi R. Saxena, Amit Dubey, Girish Chandel

**Affiliations:** ^1^ Department of Plant Molecular Biology and Biotechnology, College of Agriculture, Indira Gandhi Krishi Vishwavidyalaya, Raipur, India; ^2^ Knowledge and Technology Resource Centre, Indira Gandhi Krishi Vishwavidyalaya, Raipur, India; ^3^ Department of Vegetable Biotechnology, VNR Seeds Private Limited, Raipur, India; ^4^ Chhattisgarh Biotechnology Promotion Society, Raipur, India; ^5^ Department of Genetics and Plant Breeding, College of Agriculture, Indira Gandhi Krishi Vishwavidyalaya, Raipur, India; ^6^ Chhattisgarh Council of Science and Technology, Raipur, India

**Keywords:** transcriptome, database, Little Millet, LMT^db^, DEGs, SSRs, functional annotation, metabolic pathway

## Abstract

Little millet (*Panicum sumatrense*) a native of Chhattisgarh, belongs to the minor millet group and is primarily known as a climate-resilient and nutritionally rich crop. However, due to the lack of enough Omic studies on the crop, the scientific community has largely remained unaware of the potential of this crop, resulting in less scope for its utilization in crop improvement programs. Looking at global warming, erratic climate change, nutritional security, and limited genetic information available, the Little Millet Transcriptome Database (LMT^db^) (https://igkv.ac.in/xenom/index.aspx) was conceptualized upon completion of the transcriptome sequencing of little millet with the aim of deciphering the genetic signatures of this largely unknown crop. The database was developed with the view of providing information about the most comprehensive part of the genome, the ‘Transcriptome’. The database includes transcriptome sequence information, functional annotation, microsatellite markers, DEGs, and pathway information. The database is a freely available resource that provides breeders and scientists a portal to search, browse, and query data to facilitate functional and applied Omic studies in millet crops.

## 1 Introduction

An alarming increase in the Earth’s temperature, along with increasing water scarcity in recent years, is posing a threat to the future of global food security. In addition to this, addressing the issue of micronutrient deficiency in developing and underdeveloped nations is another formidable challenge faced by policymakers today. Enhancing the productivity and nutritional value of crops in the modern-day world is a concern that needs to be addressed efficiently to bridge the gap between demand and supply of food in the coming years. The nutritionally rich millets are low-maintenance, climate-resilient cereals that hold potential benefits for human health at minimal cost, thus providing a comprehensive solution for global food and nutritional security (Kumar et al., 2018). Nevertheless, despite the satisfaction that these crops bring to our table, sheer negligence has been observed in their cultivation and consumption (Himanshu et al., 2018). Also, the molecular aspects of stress tolerance in millets have largely remained unexplored (Babele et al., 2022).

*Panicum sumatrense* (Little millet) is one of the predominant yet lesser-studied, tetraploid species (2n = 4x = 36) of minor millets, essentially cultivated for its grain (Gomez and Gupta, 2003; Das et al., 2020). The crop is loaded with nutrients such as dietary fiber, protein, and minerals like iron (Fe = 32.20 ppm) and zinc (Zn = 32.40 ppm) (Girish et al., 2014; Vetriventhan et al., 2021) and performs exceptionally well under challenging environmental conditions and is invariably adapted to high temperature and moisture stress (Ajithkumar and Panneerselvam, 2014). Yet, to date, the crop has been considered an ‘orphan’ and has never fallen under the umbrella of extensive research until recent years. As a result of that, unlike rice, wheat, and other cereals, the crop has still not achieved breakthroughs in terms of gene discoveries, genetic improvement, and yield enhancement. Also, due to a lack of enough studies, ‘Omics’ information related to the crop has remained scarce (Natesan et al., 2020).

Transcriptomics is a promising approach that has transformed our outlook toward gene expression patterns at the cellular and molecular levels (Lowe et al., 2017). RNA-Seq, is one of the transcriptomic technologies and is known to resolve expression patterns of genes with exceptional clarity (Finotello and Di Camillo, 2015). It is evident from previous studies that, RNA-Seq has opened new opportunities for researchers and has played a pivotal role in mining a plethora of genes in a wide range of plant species, from parasitic to land plants and tree species. (Li et al., 2015; Howlader et al., 2020; Sobreiro et al., 2021). In millets as well, several genes, transcription factors, and metabolic pathways regulating biotic (Kulkarni et al., 2016) and abiotic stress tolerance (Das et al., 2020; Shivhare et al., 2020; Sun et al., 2020; Suresh et al., 2022), along with those involved in modulating metal-ion homeostasis (Chandra et al., 2020; Satyavathi et al., 2022) have also been tracked using RNA-Seq. This data-driven technology delivers enormous information upon intricate analysis and can help researchers if made publicly accessible. Foreseeing the potential of RNA-Seq data in understanding plants’ behavioral and developmental biology, scientific fraternities from all over the globe have been emphasizing to create web-based platforms which can be utilized to explore these information free of cost. FmMiRNADb, FmTFDb (Thulasinathan et al., 2022) Plant Public RNA-Seq Database (Yu et al., 2022), GRAND (Zhang et al., 2022), HpeNet (Sheng et al., 2020), TRANSNAP (Koshimizu et al., 2019), AgriSeqDB (Robinson et al., 2018), SeagrassDB (Sablok et al., 2018), and MOROKOSHI (Makita et al., 2015) are few of the popular plant expression databases developed for millets and other plants. Interestingly, even after a heedful screening, we were unable to detect any independent platform dedicated solely to little millet and this prompted us to create an interactive web-based transcriptomic resource for this non-model plant that can serve as a comprehensive guide to understanding the crop’s molecular biology. To our knowledge, the Little Millet Transcriptome Database version 1.0.0 (LMT^db^ v-1.0.0) is an exclusive, first-of-its-kind database made for little millet that runs on the Windows operating system. The database is an information-rich resource of transcriptome sequences and functions, plant metabolic networks, molecular markers, and differentially expressed genes of three tissue types, namely secondary leaf, flag leaf, and panicle under control and drought conditions, in which all data can be freely accessed for research purposes to attain global food and nutritional security.

## 2 Materials and methods

### 2.1 Illumina sequencing, assembly, and annotation

**Figure 1:**
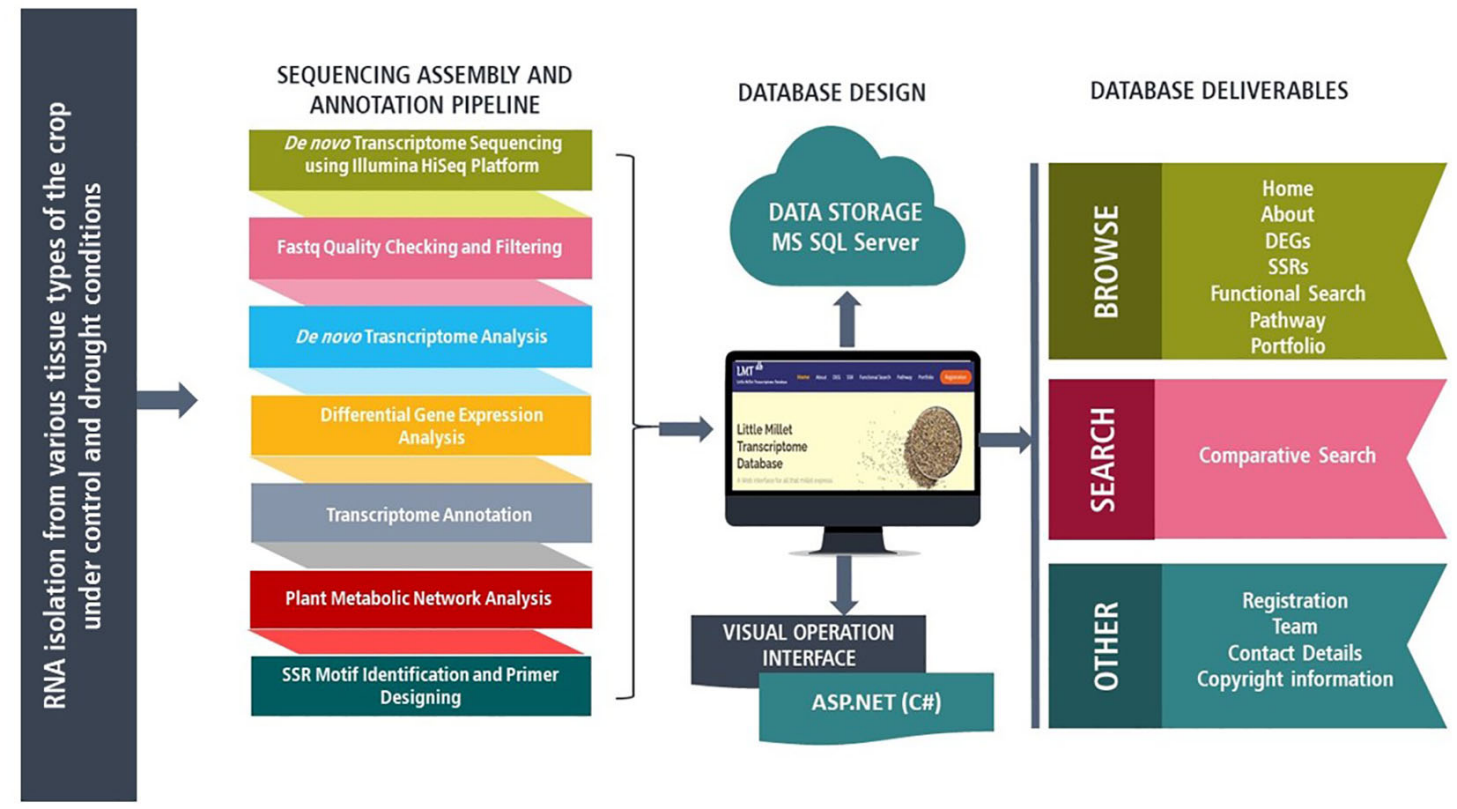
Schematics of Little Millet transcriptome database (LMT^db^ v.1.0.0) structure and web interface features along with transcriptome sequencing, assembly, and annotation pipeline.

A total of 4 samples from three different tissue types of little millet genotype RLM-37 namely secondary leaf under drought conditions and secondary leaf, flag leaf, and panicle under control conditions, were undertaken for RNA isolation. The extracted mRNAs were examined for their quality and quantity and were sequenced using the Illumina HiSeq Platform followed by quality check with the help of Trimmomatic-0.39 and *de novo* transcriptome assembly utilizing the Trinity v 2.4.0 (**Figure 1**) (Unpublished data). The unigenes of *de novo* transcriptome assembly were annotated using BLASTx (blast.ncbi.nlm.nih.gov) against NCBI non-redundant protein database (www.ncbi.nlm.nih.gov) followed by Gene Ontology mapping utilizing Blast2GO (www.blast2go.com) and motif identification using InterProScan (www.ebi.ac.uk/interpro/search/sequence/). Based on the read counts and expression values of each transcript differentially expressed genes (DEGs) were identified using edgeR. In addition, the KEGG database (www.genome.jp/kegg/) was employed for metabolic pathway annotation, and finally, MISA (www.bio.tools/misa) was undertaken for the identification of simple sequence repeats (SSRs). Also, to identify similar sequences BLASTn was performed against Reference RNA sequences (refseq_rna) database.

### 2.2 Data sources

The RNA-Seq data obtained from little millet was utilized for creating Little Millet Transcriptome Database version 1.0.0 (LMT^db^ v-1.0.0). High quality reads generated after trimming of raw reads from secondary leaf under drought conditions (SLS), and secondary leaf (SLC), flag leaf (FLC), and panicle (PC) under control conditions were undertaken for *de novo* transcriptome assembly (**Table S1**; **Table 1**). Further, after functional annotation of the assembled transcripts, a total of 128,963 unigenes were pinned down, and *via*. KEGG we identified metabolic network pathways corresponding to these unigenes. In addition, the data also includes 1,434 differentially expressed genes under control and drought conditions followed by 10,317 differentially expressed genes across three tissue types. For the identification of significant DEGs, stringent parameters such as a *p-*value (0.05), log_2_FC (± 2), and false discovery rate (FDR < 0.001) were applied. Apart from the above information, we have also identified a sum of 13,496 SSRs useful for functional genomic studies.

**Table 1:** A summary of RNA-Seq data.

Total Number of Transcripts	280,980
Total Number of Assembled Bases (all transcripts)	294,327,315
Total Number of Assembled Bases (longest isoforms per gene)	88,886850
Total Number of Genes	128,963
Percent GC	48.39
N50 (based on all transcripts)	1744
N50 (based on longest isoforms per gene)	1213

### 2.3 Designing and development of databases

The first version of the Little Millet Transcriptome Database (LMT^db^ v-1.0.0) is an MS SQL database, designed and built to run on the Windows operating system with a DOTNET framework. The proposed transcriptome database algorithms are implemented in a C# environment on a 4 core processor Intel Xeon Gold 6152 CPU remote cloud server. The standard 3 Tier architecture is used as client-server architecture for the development of the project (**Figure 1**). MS SQL server version 2017 is used as a backend for storing transcriptome sequence and annotation data. The proposed system is designed to allow its end-users to query the database through a web-based interface developed using ASP.NET, HTML5, and CSS3. The system is cleared black box test based on test cases built around the specifications and requirements of the application.

## 3 Results and discussion

The incomparable tolerance of millets toward abiotic stresses including drought, salinity, and heat along with their efficiency to sequester and load minerals from the soil to the grains make them an amenable system to understand the plant’s stress-responsive behavior and metal homeostasis at cellular, molecular and physiological levels (Bandyopadhyay et al., 2017). Next-generation sequencing approaches have been successfully harnessed to comprehend the stress and nutrition biology of both major and minor millets (Lata, 2015; Prabha et al., 2017; Pujar et al., 2020). In little millet, the *de novo* transcriptome analysis has recently been leveraged to identify genes involved in drought and salinity tolerance mechanisms (Das et al., 2020) Attempts have also been made to identify grain nutrient related and moisture stress-responsive gene orthologous (Chandel et al., 2017; Sushmitha et al., 2018) and genic microsatellite markers in little millet (Desai et al., 2021). Further, the chloroplast genome of little millet has been sequenced and employed to investigate the crop’s phylogenetic relationship with other members of Poaceae (Sebastin et al., 2018). However, the crop is still undermined as not much is known about molecular mechanisms that govern metal homeostasis and several other functions.

It is noteworthy that over the past decade gene discovery *via* transcriptomic approaches has been much more frequently observed than that through genomic approaches. Transcriptomics allows us to track changes in gene expression patterns over tissue types, environmental conditions, and developmental stages with great precision. With this idea, several researchers across the globe have developed open-access transcriptomic portals or databases for minor millets which are currently being explored for mining genes associated with biotic and abiotic stress tolerance, metal homeostasis, and also for yield and quality improvement. However, to our knowledge, no such unified browsing platform has been specifically designed and developed for little millet posing restrictions in employing forward genetic approaches to penetrate deeper into the molecular architecture of the crop. To bridge this gap, we developed the Little Millet transcriptome database (https://igkv.ac.in/xenom/index.aspx), encompassing extensive information on transcriptome assembly and annotation of little millet under one umbrella.

### 3.1 Architecture of LMT^db^

**Figure 2:**
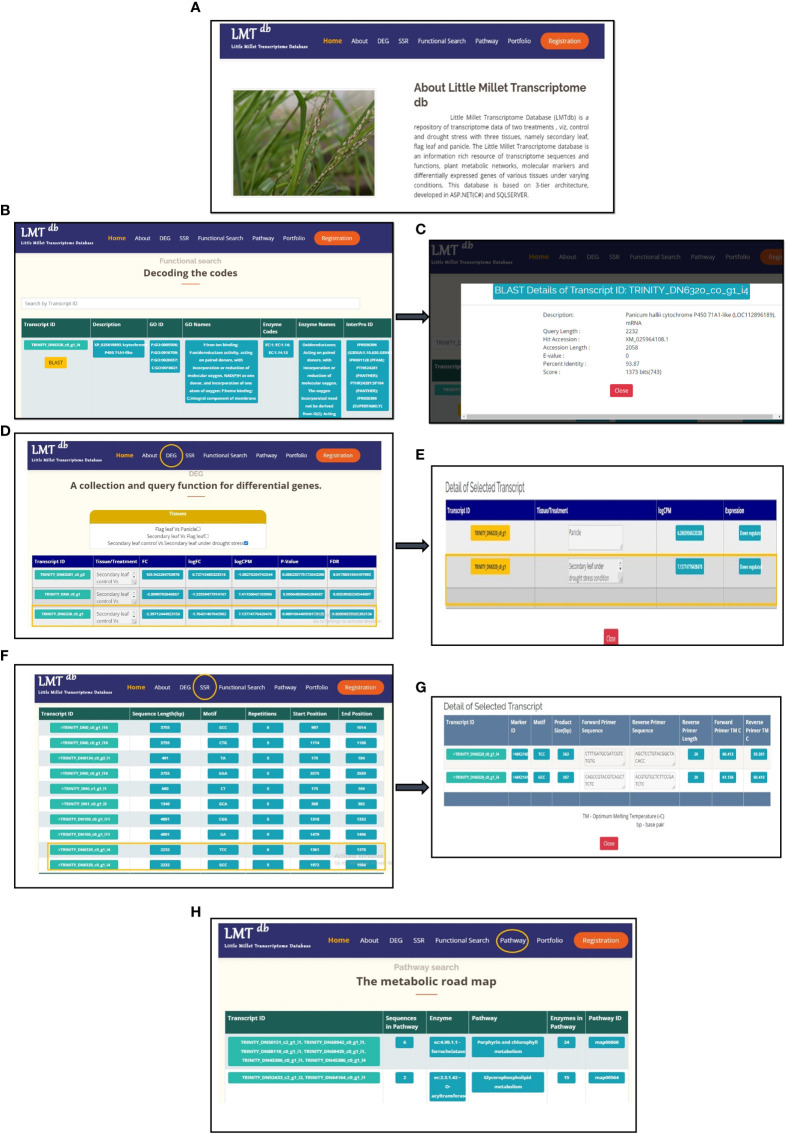
Schematic representation and screenshots of Little Millet transcriptome database (LMT^db^ v.1.0.0). **(A)** Representation of key elements (DEGs, SSRs, Functional Search, Pathway, and Portfolio) of the database as displayed on the database homepage. **(B)** Functional Search page displaying information related to CYP71A1 like gene (TRINITY_DN6320_c0_g1_i4). **(C)** BLAST details for CYP71A1 like gene (TRINITY_DN6320_c0_g1_i4) **(D)** Comparative search for CYP71A1 like gene under DEG module. **(E)** Downregulation of CYP71A1 like gene in secondary under drought stress. **(F)** Representation of SSRs information for CYP71A1 like gene. **(G)** Primer details corresponding to CYP71A1 like gene. **(H)** Metabolic pathway information with enzymes and pathway IDs corresponding to different transcripts.

LMT^db^ v-1.0.0 is a compendium of the expressed parts of the little millet’s genome. It has a comprehensive yet methodical representation of transcriptome data and has been proficiently designed to provide absolute ease to its end-users while browsing and searching for desired information in the database (**Figure 1**). The database encompasses transcriptome information for three different tissue types (secondary leaf, flag leaf, and panicle) under drought and control conditions. It also provides a brief description of the tools and frameworks that have been utilized for designing the database which shall guide researchers willing to design a similar kind of repository for some other crop. Structurally, the database comprises eight key tabs namely: Home, About, DEGs, SSRs, Functional Search, Pathway, Portfolio, and Registration (**Figure 2A**). The ‘home’ page has been designed in a way that makes browsing effortless and intuitive for its end-users, with key elements of the database being accessible from all the relevant locations throughout the home page. As LMT^db^ is one of the first databases designed precisely for little millet, we have also provided a brief description regarding the crop and its importance in the modern world in the ‘About’ section of the website. The platform provides browse and search functions for transcriptomic information including, gene expression profiles (DEGs) genomic variations (SSRs), and functional and metabolic pathway annotation.

### 3.2 Browse functions in LMT^db^

The transcriptomic information stored in the database is freely accessible to the public and can be browsed under the module names DEGs, SSR, Functional Search, Pathway, and Portfolio on the database home page. The corresponding information for each of these modules is represented in a tabular format and can be visualized upon clicking directly on the module name. The data under the module DEG can be browsed under seven key attributes: a unique transcript identifier as Transcript ID, Tissue type or Treatment information, Fold Change (FC) value; Logarithm of Fold Change (logFC), Logarithm of Counts Per Million Reads (logCPM), Probability value (*p-*value) and False Discovery Rate (FDR) (**Figure 2D**). Information related to gene expression patterns (upregulation or downregulation) can be retrieved by clicking through Transcript IDs (**Figure 2E**). This information can further be analyzed to obtain a clear picture of candidate genes involved in stress tolerance and also for various developmental processes of the plant. In a similar fashion information about genomic variations i.e. SSRs can be browsed under 6 attributes, namely: Transcript ID, Sequence Length (in base pairs), SSR Motifs (classified based upon motif length as di, tri, tetra, penta, hexa, hepta, octa, and nona), Motif Repetition, and Start and End Positions of each marker (**Figure 2F**). Primer details, including marker ID, forward and reverse primer sequences in base pairs along with their melting temperatures and product size in base pairs can be accessed by clicking on the desired transcript IDs (**Figure 2G**). This information can be employed to track SSRs based genetic variation across species and also for marker-trait association studies for crop improvement. Apart from these, one can browse through the Functional Search and Pathway tabs to gain insights into the molecular functions and metabolic network pathways of the assembled transcripts. The functional search depicts the information related to functions of the transcript sequences under the attributes: Description, GO IDs, GO Names, Enzyme Codes, Enzyme Names, and InterPro, inclusive of Panther, Prosite, Gene3D, and Pfam IDs (**Figure 2B**). In addition to this, the functional search tab also provides BLAST details for given transcripts, which can be retrieved upon clicking the BLAST option (**Figure 2C**). Similarly, the Pathway tab displays information regarding plant metabolic networks under the attributes: Pathway Name, Pathway IDs, EC Number of enzymes, and Total Number of Enzymes involved in each respective pathway (**Figure 2H**). Users can access detailed pathway information from the KEGG database (https://www.genome.jp/kegg/) using the pathway IDs provided. Additionally, the database also has a portfolio section that serves as a collection of pathway maps, heat maps, and gene distribution graphs obtained as an outcome of transcriptome analysis. The section has been curated to make data interpretations visually appealing and uncomplicated for our end-users.

### 3.3 Search functions and retrieval of information in LMT^db^

LMT^db^ allows users to perform a comparative search under the DEGs module. A web interface for DEGs is divided into two sections, the filter and the result section (**Figure 2D**). The filter section allows filtering comparative data by checking or unchecking the check box. The comparative search options comprise (i)Flag leaf vs. Panicle under control condition, (ii) Secondary leaf vs. Flag leaf under control condition, and (iii) Secondary leaf under control condition vs. Secondary leaf under drought stress condition. Based upon comparative search, the data will be updated in the result section in tabular format. Users after making a registration (https://igkv.ac.in/xenom/index.aspx) can retrieve transcriptome information free of cost. Apart from this, ‘Search by Transcript ID’ allows feasible exploration of robust data-set based on transcript IDs.

### 3.4 Applications of LMT^db^

#### 3.4.1 Case study of a drought-responsive gene

Plants under drought or dehydration stress elicit several enzymatic and hormonal responses involved in ROS scavenging, ion uptake and transportation, and accumulation of osmoprotectants (Shinozaki and Yamaguchi-Shinozaki, 2007). Cytochrome P450s (CYPs) are a member of the class oxidoreductase and are known to be one of the largest gene families in angiosperms. CYPs catalyze NADPH-and or/O2-dependent hydroxylation reactions in living systems and have been extensively studied in various plant species with an aim to discern their roles in response to biotic and abiotic stress (Pandian et al., 2020).

**Figure 3:**
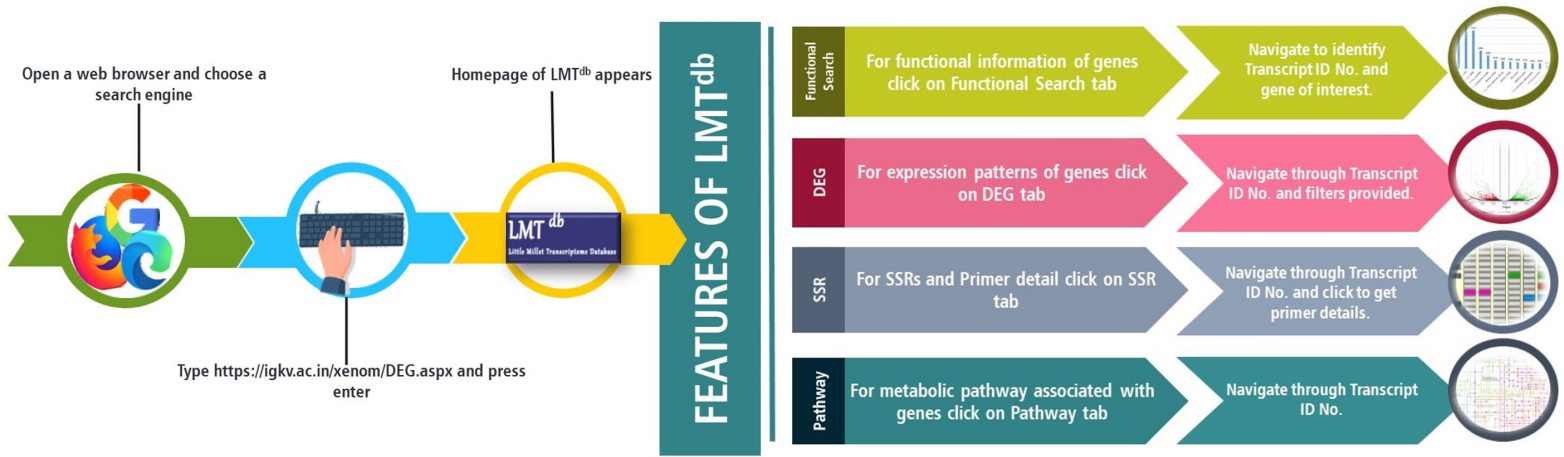
Schematics regarding access, browse and search gene functions in Little Millet transcriptome database (LMT^db^ v.1.0.0).

To demonstrate the applications of the current version of LMT^db^, we performed a case study undertaking CYP71A1 gene from the Cytochrome P450 superfamily. The CYP71A1 gene codes for tryptamine 5-hydroxylase enzyme involved in plant serotonin/melatonin biosynthesis pathway (Bhowal et al., 2021). In the past few years, the gene has reportedly been studied to understand biotic (Lu et al., 2018), and abiotic stress tolerance (Zhang et al., 2012) in rice. To assess the role of CYP71A1 gene in little millet under drought stress, we tried exploring LMT^db^. To decipher the function of this gene, the functional search option on the database home page was carefully screened, and the putative function of CYP71A1 like gene along with its unique transcript ID (TRINITY_DN6320_c0_g1_i4) was located (**Figure 2B**). BLAST details for the given transcript showed the highest similarity with Panicum hallii cytochrome P450 71A1-like mRNA with a percent identity of 93.87%, and a score of 1373 bits(743) (**Figure 2C**). This transcript ID was then used to screen the ‘DEG’ module under secondary leaf control and drought stress comparative search option. Under the DEG module, downregulation of CYP71A1 gene (logFC -1.76431407043982 and *p*-value 1.06E-0) in secondary leaf under drought conditions was observed (**Figures 2D, E**). Further, we also identified two different SSRs, each with one primer, for the given transcript ID (**Figures 2F, G**). A similar workflow can be utilized by researchers for tracking transcriptomic information related to their genes of interest (**Figure 3**).

#### 3.4.2 A comparative study of LMT^db^ with MOROKOSHI databases

**Figure 4:**
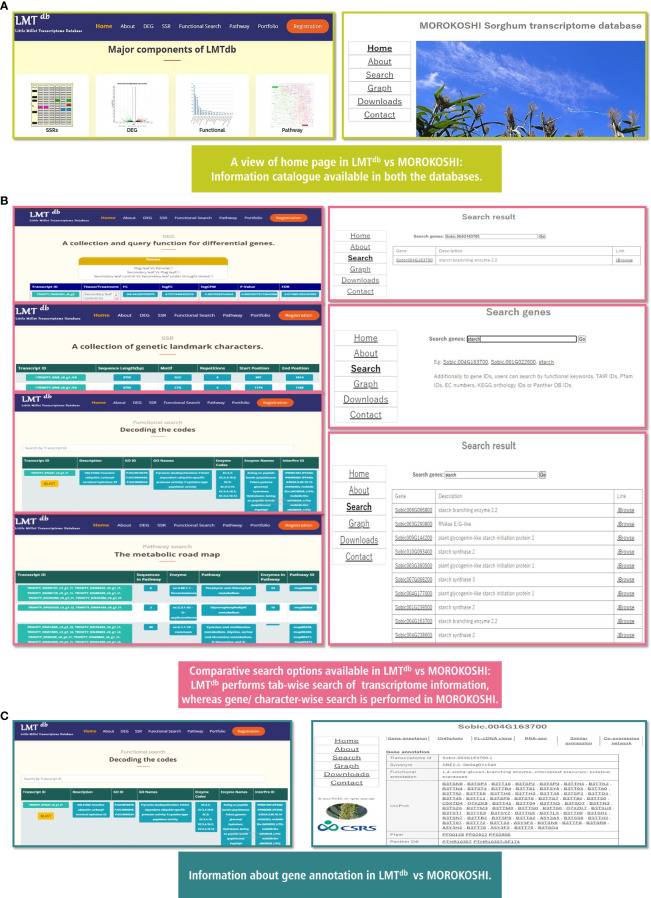
Comparative representation of Little Millet transcriptome database (LMT^db^ v.1.0.0) and MOROKOSHI database. **(A)** A view of home page in Little Millet Transcriptome Database (LMT^db^ v.1.0.0) vs MOROKOSHI Database. **(B)** Comparative search options available at Little Millet Transcriptome Database (LMT^db^ v.1.0.0) vs MOROKOSHI Database. **(C)** Information about gene annotation in Little Millet Transcriptome Database (LMT^db^ v.1.0.0) vs MOROKOSHI Database.

To understand the relevance of LMT^db^ v.1.0.0 for millet research in the current scenario, we compared our database with one of the advanced databases in millet i.e. MOROKOSHI: pTranscriptome Database in Sorghum bicolor (Makita et al., 2015) (**Figure 4**) (http://sorghum.riken.jp/Home.html). The look and feel of MOROKOSHI seems quite similar to our database with key tabs featuring Home, About, Search, Graph, Download, and Contact (**Figure 4A**), and the transcriptome information in both databases appear as static web pages. However, a major difference in terms of browse and search functions can be seen between both databases. In LMT^db^, transcriptome information is available in a tab-wise (DEG, SSR, Pathway, and Functional Search) manner whereas, in MOROKOSHI gene/character-wise information is available (**Figure 4B**). It is noteworthy that, both the databases are at par in terms of outcomes of gene annotation comprising functional annotation, Uniprot, Pfam, Panther, GO, and KEGG IDs for the genes (**Figure 4C**). Nevertheless, there are a few differences that we encountered while comparing the two. LMT^db^ provides a comprehensive detail of microsatellite markers which is apparently not available in MOROKOSHI. On the contrary, MOROKOSHI provides additional features like orthologs, RNA-Seq expression profile, and co-expression network which we are yet to add in LMT^db^. Another key difference that we noticed was MOROKOSHI runs on a Linux-based OS platform whereas, LMT^db^ uses a Windows-based OS platform for the same. Although MOROKOSHI provides more information with respect to analysis and cataloging compared to LMT^db^ it is crucial to note that little millet is still understudied compared to sorghum. We expect that with the additional data to be added shortly, we can upgrade the information content of our database in near future in the upcoming version.

### 3.5 Limitations

The current version of LMT^db^ has some limitations for example, the database lacks hyperlinks to other web-based bioinformatic tools and we have planned to upgrade this in upcoming versions of the database.

## 4 Conclusion and future perspectives

LMT^db^ provides its end-users a web interface to access various information related to the expressed part of little millet’s genome, such as DEGs, microsatellite markers, gene functions, and plant metabolic pathway information. The above data can be browsed, analyzed, and even downloaded upon registration, free of cost, and can contribute to functional as well as comparative Omic studies elucidating evolutionary and developmental biology of the crop. The information in this database is highly useful for the identification and characterization of novel genes, understanding the drought and tissue-specific gene expression patterns, and also in designing SSR-based fingerprinting studies in little millet as well as in other related minor millets. The database will serve as a comprehensive resource for breeders and scientists involved in addressing global food and nutritional security through crop improvement programs. In the forthcoming versions of LMT^db^, hyperlinks to various bioinformatic tools will be added along with information related to SNP, Indels, protein-protein interaction, and gene families making it an even more extensive platform for Omic studies.

## Data availability statement

The original contributions presented in the study are publicly available. This data can be found here: NCBI accession PRJNA718414.

## Author contributions

GC conceptualized the idea. GC, AP, SS, KB, AK, and RS curated, and formatted the data and worked on the design and development of the database. AS provided the experimental material. GC AM, MD, ArP, VP, and AV carried out the preliminary wet-lab experiments and data analysis. GC, AP, SS, KB, AK, and RS drafted the original manuscript. All authors contributed to the article and approved the submitted version.
