# Supplementary material for: LMTdb: A comprehensive transcriptome database for climate-resilient, nutritionally rich little millet (Panicum sumatrense)
Source: Front Plant Sci. 2023 Mar 9;14:1106104. doi: 10.3389/fpls.2023.1106104 (PMC10041709; doi:10.3389/fpls.2023.1106104)
Supplement: Supplementary file 1 [file Table_1.docx]

Supplementary Material

LMT^db^: A comprehensive transcriptome database for climate-resilient, nutritionally rich little millet (*Panicum sumatrense*)

Shweta Shekhar^1†^, Archana S. Prasad^1†^, Kalpana Banjare^2†^, Abhijeet Kaushik^2†^, Ajit K. Mannade^1^, Mahima Dubey^3^, Arun Patil^3^, Vinay Premi^1^, Ashish K. Vishwakarma^4^, Abhinav Sao^5^, Ravi R. Saxena^2†^, Amit Dubey^6^ Girish Chandel^1†*^

*** Correspondence:**Girish Chandel

ghchandel@gmail.com

**Table S1** Quality check result summary of RNA Sequencing Reads for little millet tissue types under drought and control conditions included in this study.

| **Sample**  **Name** | **Input Reads** | **Surviving Reads** | **Percentage of Surviving Reads** |
| --- | --- | --- | --- |
| SLS1 | 34,659,323 | 32,694,484 | 94.33% |
| SLS2 | 39,512,010 | 37,481,894 | 94.86% |
| SLC1 | 50,864,529 | 48,259,604 | 94.88% |
| SLC2 | 45,557,488 | 41,968,945 | 92.12% |
| FLC1 | 49,531,202 | 47,456,301 | 95.81% |
| FLC2 | 47,086,342 | 45,212,160 | 96.02% |
| PC1 | 43,076,907 | 42,455,756 | 98.56% |
| PC2 | 40,332,952 | 39,422,823 | 97.74% |

SLS: Secondary leaf under drought conditions

SLC: Secondary leaf under control condition

FLC: Flag leaf under control condition

PC: Panicle under control condition
